# Rural and Ethnic Disparities in Out-of-hospital Care and Transport Pathways After Road Traffic Trauma in New Zealand

**DOI:** 10.5811/westjem.18366

**Published:** 2024-06-04

**Authors:** Rebbecca Lilley, Gabrielle Davie, Bridget Dicker, Papaarangi Reid, Shanthi Ameratunga, Charles Branas, Nicola Campbell, Ian Civil, Bridget Kool

**Affiliations:** * University of Otago, Dunedin School of Medicine, Department of Preventive and Social Medicine, Injury Prevention Research Unit, Dunedin, New Zealand; † Auckland University of Technology, Department of Paramedicine, Faculty of Health and Environmental Sciences, Auckland, New Zealand; ‡ Hato Hone St John, Mt Wellington, Auckland, New Zealand; § Waipapa Taumata Rau-University of Auckland, Te Kupenga Hauora Māori, Faculty of Medical and Health Sciences, Auckland, New Zealand; ∥ University of Auckland, School of Population Health, Section of Epidemiology and Biostatistics, Auckland, New Zealand; ¶ Te Whatu Ora (Health New Zealand) Counties Manukau, Population Health Directorate, Auckland, New Zealand; # Monash University, Department of Epidemiology and Preventive Medicine, Faculty of Medicine Nursing and Health Sciences, Melbourne, Australia; ** Columbia University Mailman School of Public Health, Department of Epidemiology, New York, New York; †† Auckland District Health Board, Trauma Services, Auckland, New Zealand

## Abstract

**Introduction:**

The out-of-hospital emergency medical service (EMS) care responses and the transport pathways to hospital play a vital role in patient survival following injury and are the first component of a well-functioning, optimised system of trauma care. Despite longstanding challenges in delivering equitable healthcare services in the health system of Aotearoa-New Zealand (NZ), little is known about inequities in EMS-delivered care and transport pathways to hospital-level care.

**Methods:**

This population-level cohort study on out-of-hospital care, based on national EMS data, included trauma patients <85 years in age who were injured in a road traffic crash (RTC). In this study we examined the combined relationship between ethnicity and geographical location of injury in EMS out-of-hospital care and transport pathways following RTCs in Aotearoa-NZ. Analyses were stratified by geographical location of injury (rural and urban) and combined ethnicity-geographical location (rural Māori, rural non-Māori, urban Māori, and urban non-Māori).

**Results:**

In a two-year period, there were 746 eligible patients; of these, 692 were transported to hospital. Indigenous Māori comprised 28% (196) of vehicle occupants attended by EMS, while 47% (324) of patients’ injuries occurred in a rural location. The EMS transport pathways to hospital for rural patients were slower to reach first hospital (total in slowest tertile of time 44% vs 7%, *P* ≥ 0.001) and longer to reach definitive care (direct transport, 77% vs 87%, *P* = 0.001) compared to urban patients. Māori patients injured in a rural location were comparatively less likely than rural non-Māori to be triaged to priority transport pathways (fastest dispatch triage, 92% vs 97%, respectively, *P* = 0.05); slower to reach first hospital (total in slowest tertile of time, 55% vs 41%, *P* = 0.02); and had less access to specialist trauma care (reached tertiary trauma hospital, 51% vs 73%, *P* = 0.02).

**Conclusion:**

Among RTC patients attended and transported by EMS in NZ, there was variability in out-of-hospital EMS transport pathways through to specialist trauma care, strongly patterned by location of incident and ethnicity. These findings, mirroring other health disparities for Māori, provide an equity-focused evidence base to guide clinical and policy decision makers to optimize the delivery of EMS care and reduce disparities associated with out-of-hospital EMS care.

Population Health Research Capsule What do we already know about this issue?

*Poorer injury outcomes in rural, indigenous, and minority communities are well documented.*

What was the research question?

*What are the rural and ethnic inequities in out-of-hospital care and transport pathways following traffic crashes in New Zealand?*

What was the major finding of the study?

*Disparities were most evident in rural Māori: less likely to first be transported to (33 vs 56%, p < 0.001), or ever reach a tertiary care hospital (51 vs 73%, p < 0.001).*

How does this improve population health?

*More equity-focused planning and investment in rural EMS services to reduce documented disparities in EMS care would benefit both rural and indigenous populations.*

## INTRODUCTION

Recent decades have seen the evolution of out-of-hospital emergency medical services (EMS) from transportation of patients to emergency departments (ED) through to clinicians of advanced out-of-hospital healthcare and delivery of major trauma patients directly to appropriate care via a range of transportation means and destination pathways.^1^ These EMS responses and the transport pathways to hospital play a key role in patient survival and are the first component of a well-functioning, optimised system of trauma care. Internationally there is growing recognition of the critical need to eliminate inequities in healthcare. Poorer outcomes following major injury for residents of rural communities and for indigenous and minoritized ethnic groups are well documented,^2^ with evidence of longer times to reach definitive care for rurally located injured patients^3^^–^^6^ and lower standards of EMS care and transport for racial and ethnic minorities.^7^ However, little is known about differential access to or delivery of out-of-hospital EMS care for rural and ethnic sub-groups, in particular whether disparities in trauma outcomes can be reduced by more equitable access to EMS care and designated transport pathways.

Population-level data on EMS-delivered out-of-hospital care and transport pathways to hospital can help inform the optimisation of national EMS systems, address inequities, and improve patient outcomes following major trauma, yet major knowledge gaps remain in these areas. The national healthcare system of Aotearoa New Zealand (NZ) has had longstanding challenges in delivering equitable levels of access to healthcare services to indigenous Māori and to rural communities.^8^^–^^10^ Māori, as indigenous people of Aotearoa, are partners to the health equity commitments under Te Tiriti – Treaty of Waitangi with the Crown, yet they experience pervasive inequities.^11^ Previous research has identified longer theoretical access times to out-of-hospital EMS care for Māori, which are hypothesized to reflect, in part, the higher proportion of Māori residing in rural regions with limited timely access to healthcare services.^12^^,^^13^ Improvements in trauma outcomes, therefore, require investigation of inter-related inequities based on both geography and ethnicity. This major gap in knowledge is reflected in the national EMS systems of other nations with comparable health system contexts and similarly situated rural remote and indigenous populations, thereby further motivating the need for investigation.

The actual out-of-hospital EMS care responses and transport pathways to hospital experienced by under-served rural and Māori populations and the interconnected and overlapping geographic and ethnic disparities remain unexplored at a national level. Deeper understanding of sources of disparities in EMS care and transport pathways to hospital are the first step in guiding quality improvements and planning for equitable out-of-hospital EMS services. Our objective in this analysis was to describe potential geographic, and intersectional geographic and ethnic inequities, in out-of-hospital care and the transport pathways to hospital delivered by NZ EMS professionals following major trauma due to road traffic crashes (RTC).

## METHODS

### Study Design and Setting

In this observational study we used a retrospective cohort based on two years (2016–2018) of clinical and EMS utilisation data from NZ’s two road ambulance services: Hone Hato St John, servicing 97% of NZ’s geographical area; and Wellington Free Ambulance, servicing the remaining greater Wellington and Wairarapa. Data is routinely collected in a prescribed format by ambulance staff to create a collective electronic administrative resource comprised of individual electronic patient report forms (ePRF); this objective data was used for analysis. The full study protocol has been published elsewhere.^14^^,^^15^

Out-of-hospital EMS are predominantly based on the provision of emergency road ambulance services. Road services are predominantly dispatched in the first instance. Air services, operating helicopters on a regional basis, are dispatched on an as-needed basis to provide additional clinical care to access remote sites or facilitate timely transport of seriously injured patients. Emergency medical serrvices are readily accessible via a single, national emergency telephone number (111) with two national ambulance control centres triaging and dispatching appropriate EMS. Funding for EMS services provided within 24 hours of an injury incident is covered by NZ’s universal no-fault injury provider, the Accident Compensation Corporation.^16^

New Zealand’s trauma system, covering the two main islands of 265,000 km^2^ and approximately five million people, is designed around four regional nodes of trauma care with 22 trauma-receiving hospitals.^17^^,^^18^ Each node has at least one metropolitan, tertiary trauma hospital service providing intensive care, advanced resources, and services around the clock, generally similar to Level 1 American College of Surgeons-verified trauma centres.^19^ Regional trauma hospitals are capable of initial resuscitation, stabilisation, intensive care and, in some instances, definitive management of injured patients. Small rural hospitals are capable of basic non-specialist trauma services with limited trauma specialisation and resources.^18^^,^^20^

The New Zealand Major Trauma Destination Policy, which is applied in out-of-hospital trauma responses, was introduced in 2017 to improve survival from major trauma.^21^ The policy outlines the eligibility criteria to be assessed by EMS professionals at the scene for direct transfer to a major trauma centre.^20^

### Selection of Participants

To obtain a dataset of EMS-attended major trauma patients, we undertook linkage between electronic records of EMS attendance and the New Zealand Trauma Registry (NZTR), a registry of all hospitalised major trauma patients.

Study participants were individuals aged 0–84 years who had suffered a major trauma as defined by the NZTR (Injury Severity Score, [ISS] >12, or died in or out-of-hospital) and had been attended by a road EMS professional between 1 December 2016–30 November 2018. Attendance by air EMS professionals was captured in the records of attendance taken by road EMS professionals. We excluded patients with incomplete clinical records. For this analysis study participants were restricted to motor vehicle occupants who sustained injuries during a RTC to allow for any inequities in EMS care to be identified irrespective of differences in injury mechanism. To focus on those patients with the most to benefit from timely EMS care and transport, analyses were conducted on patients assessed by ambulance staff as having an on-scene EMS triage condition of status 1 (critical, immediate threat to life) or status 2 (serious, potential threat to life). Analyses describe all non-transported (ie, died on scene, refused transport) and transported patients, and then focus on EMS pathways by restricting analyses to those transported from the scene by EMS.

### Measurements

We obtained sociodemographic characteristics of age, gender, and ethnicity from the Ministry of Health’s National Health Index database. Ethnicity is collected in national health data using established data collection protocols and allows for people to self-identify up to three separate ethnic affiliations. In accordance with Te Tiriti principles and ethnicity data protocols in NZ,^22^ ethnicity was categorised as Māori and non-Māori, prioritising Māori if any of the Ministry of Health-recorded ethnicity fields were Māori.

The geographic location of injury incident was determined by applying the 2018 Geographical Classification for Health (GCH) to EMS-recorded co-ordinates of the patient’s location; the two major- level GCH classifications of rural or urban was used.^23^ We determined the “rurality” of the injury incident by applying the 2018 GCH to EMS-recorded co-ordinates of the patient’s location; the two major-level GCH classifications of rural or urban (includes suburban) were used.^23^ We used population, drive-time thresholds, and stakeholder workshops to classify small areas into GCH categories, which were then validated quantitatively. Injury characteristics included dominant injury type (blunt or penetrating) and presence of traumatic brain injury as assessed on scene by EMS staff. The NZTR provided data on ISS, which is automatically coded using Abbreviated Injury Scale codes entered at hospital discharge. We classified ISS values into two groups: survivable (ISS ≤ 25) and reduced survivability (ISS > 25).

**Figure 1.**
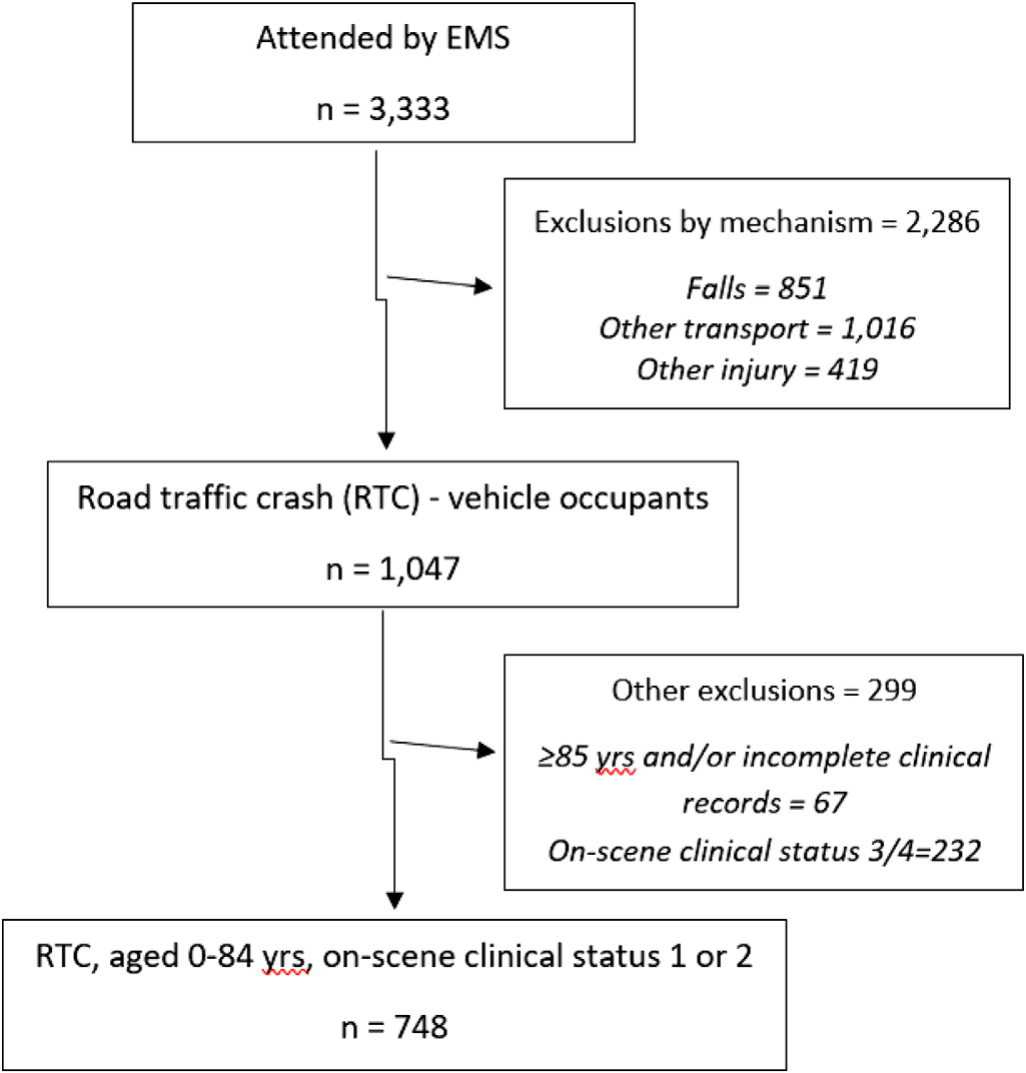
Definition of life-threatening event and out-of-hospital interventions (consistent with Gomes et al, 2010).^24^ *EMS*, emergency mdical servics; *CPR*, cardiopulmonary resuscitation.

We determined on-scene patient status and vital signs from EMS staff data. The Glasgow Coma Scale (GCS) indicates the degree of patient consciousness ranging from entirely unresponsive (scored 3) to normal response (scored 15): categorised ≤10 and >10. Pulse rate was grouped into one of two categories: 60-130 beats per minute or “<60 or >130.” Systolic blood pressure was dichotomised: <90 and ≥90 millimeters of mercury. Life-threatening events that could jeopardise patient survival were defined using the methodology of Gomes et al (2010).^24^ We identified these events using EMS clinical impressions captured on scene and grouped them into airway (A), breathing (B), circulation (C), and neurological disability (N) based on the commonly used ‘Airway Breathing Circulation’ approach for identifying and treating life-threatening events following trauma (Figure 1).^25^^,^^26^

### Outcome Measures

Outcome measures of EMS care and transport used in this study were predominantly captured in emergency road ambulance staff data, which we categorised as follows.

*Measures of EMS infrastructure and practice level at incident* included the highest practice level of crew attending the incident categorised into three categories reflecting the increasing level of skill of EMS staff on scene: emergency medical technician (EMT), paramedic, and intensive care paramedic (ICP). A variable indicating whether a single vehicle crew attended the incident was generated from EMS vehicle attendance count.

*EMS transport pathways to trauma care* included measures of final computer-aided dispatch triage status as assigned by the EMS professional, direct transport to highest level of hospital care during the care episode, and whether transport involved air ambulance. We also included the level of trauma care of the first receiving hospital (level 1 [L1] being the highest level in NZ), and whether the patient reached a tertiary trauma hospital (L1) during the episode of care. Total time to reach hospital was grouped according to the overall distribution of this variable, with the slowest tertile (ie, slowest third) corresponding to total times ≥113 minutes. We calculated theoretical access time to hospital-level care (categorised into <60 minutes, ≥60 minutes); this measure captures the estimated shortest time taken to travel from the road ambulance base location to the locations of the incident, and then to the hospital location.^27^

*EMS interventions* delivered to address life-threatening events identified in the patient on scene were identified and classified using a modified version of classification from Gomes et al (2010)^24^ (Figure 1).

We created aggregate measures of ‘any life-threatening event’ and ‘any out-of-hospital intervention received’. Unmet need was measured by identifying those with a life-threatening event who received no out-of-hospital intervention on scene.

### Primary Data Analysis

Analyses describe the transport status for the total cohort and the patterns of EMS care received and transport pathways for the transported sub-cohort receiving EMS care, using frequencies and proportions. We used chi-squared tests to compare proportions, with *t*-tests used to compare means between those injured in different geographical locations (rural/urban) and between those in combined ethnicity-geographical locations (rural Māori/rural non-Māori and urban Māori/urban non-Māori). Following the advice of Rothman,^28^^,^^29^ no adjustment was made for multiple comparisons. Instead, *P* values have been provided to sufficient precision, so that readers can apply a threshold for significance if they wish.^30^ Statistical analyses were performed using Stata SE, version 17 (StataCorp, College Station, TX).^31^

## RESULTS

### Characteristics of Study Subjects

**Figure 2.**
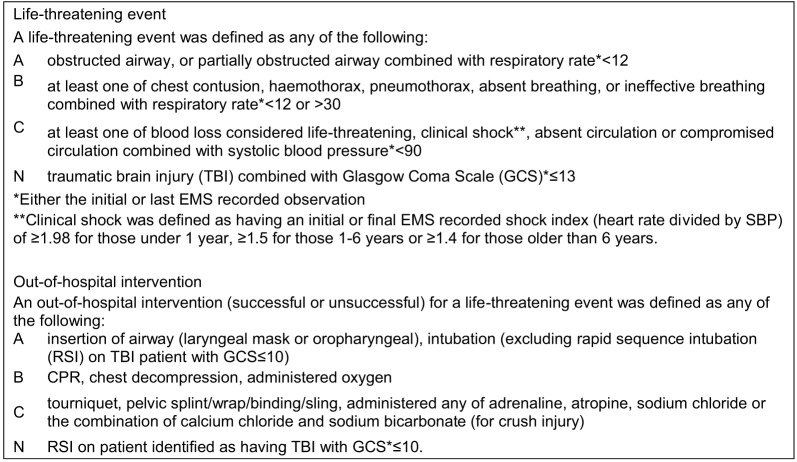
Flow diagram of vehicle occupant cohort selection.

The study population was comprised of 3,333 patients attended by an out-of-hospital EMS professional; of these, 748 met the inclusion criteria (Figure 2). A total of 56 patients in this cohort were not transported: one who declined transport and 55 patients who died on scene (Table 1). There was no evidence of differences in the distribution of on-scene deaths by location of incident or ethnicity. However, when compared to the overall proportion of Māori in the NZ population (17% of the NZ population aged ≤85 years^17^), Māori were disproportionately represented amongst on-scene fatalities due to RTC (19/55, 36% of on-scene RTC fatalities, χ^2^ = 4.82 *P* = 0.03). Of those meeting the criteria, 692 (93%) were transported to a hospital by an EMS professional and are described further.

**Table 1.** Emergency medical services transport status of road-traffic crash vehicle occupant cohort, by incident location and ethnicity (n = 748).

					Combined incident location and ethnicity					
		Incident location			Rural			Urban		
	Total (n = 748)	Rural (n = 345)	Urban (n = 397)		Māori (n = 93)	non-Māori (n = 250)		Māori (n = 120)	non-Māori (n = 270)	
	n (%)	n (%)	n (%)	*P*-value	n (%)	n (%)	*P*-value	n (%)	n (%)	*P*-value
Transported	692 (92.6)	324 (93.9)	364 (91.7)		85 (91.4)	239 (95.6)		109 (90.8)	253 (93.7)	
Died on scene	55 (7.3)	21 (6.1)	33 (8.1)	0.3	8 (8.6)	11 (4.4)	0.1	11 (9.2)	17 (6.3)	0.3
Declined transport	1 (0.1)									

Missing items: Of those transported, 4 patients are missing incident location, and 8 patients are additionally missing ethnicity. Of those who died on scene, 1 patient is missing incident location, and 7 patients are additionally missing ethnicity.

The transported cohort had a mean age of 42 years and was predominantly male (59%) (Table 2). Indigenous Māori comprised 28% (196 patients) of status 1 and 2 vehicle occupants attended by EMS, while 47% (324) of patients’ injuries occurred in a rural location. All injuries sustained by vehicle occupants were classified as blunt injuries. Differences in patient demographics and on-scene clinical status in the cohort were evident for Māori (Table 2). Rural Māori were on average four years younger while urban Māori were 11 years younger compared to their non-Māori counterparts. Despite similar average ISS scores and proportions of very severe ISS scores (ISS ≥ 25), on-scene EMS clinical triaging assessments differed markedly. However, ISS is calculated post event and is not available on scene to inform clinical triaging assessments by ambulance staff. A higher proportion of rural non-Māori patients were clinically assessed as having “potentially life-threatening” injuries (79% vs 68% of rural Māori, *P* = 0.03) while the opposite was observed in the urban setting (73% of urban non-Māori vs 84% of Māori, *P* = 0.01). The incidence of an assessment of GCS ≤ 13, indicating moderate to severe brain injury, was higher in urban Māori patients (16% vs 9% in urban non-Māori, *P* = 0.05).

**Table 2.** Patient demographics, injury characteristics and patient status on scene, by incident location and ethnicity (n = 692).

					Combined incident location and ethnicity					
		Incident location			Rural			Urban		
	Total (692)	Rural (324)	Urban (364)		Māori (85)	non-Māori (239)		Māori (109)	non-Māori (253)	
	Mean	Mean	Mean	*P*-value	Mean	Mean	*P*-value	Mean	Mean	*P*-value
Mean age	42.42	41.59	43.17	0.8	38.41	42.8	0.03	35.08	46.49	<0.001
Mean ISS	19.47	19.48	19.49	0.5	19.18	19.59	0.3	19.96	19.27	0.8
	n (%)	n (%)	n (%)		n (%)	n (%)		n (%)	n (%)	
Male	409 (59)	190 (59)	217 (60)	0.7	54 (63)	136 (57)	0.2	69 (69)	147 (58)	0.3
Māori	196 (28)	85 (26)	109 (30)	0.2			–			–
Rural	324 (47)			–			–			–
TBI	50 (7)	19 (6)	31 (8)	0.1	6 (7)	13 (5)	0.5	9 (8)	22 (84)	0.8
ISS >25	128 (18)	61 (19)	67 (18)	0.9	16 (19)	45 (19)	0.9	24 (22)	42 (17)	0.2
Immediate threat to life	530 (77)	248 (76)	278 (76)	1	58 (68)	190 (79)	0.03	92 (84)	186 (74)	0.01
Systolic blood pressure (<90 mm Hg)	23 (3)	13 (4)	9 (2)	0.2	5 (6)	8 (3)	0.3	3 (3)	6 (2)	0.8
GCS (≤13)	82 (12)	40 (12)	42 (11)	0.7	10 (12)	30 (13)	0.8	18 (16)	24 (9)	0.05
Pulse (<60 or >130 bpm)	43 (6)	25 (8)	18 (5)	0.1	10 (12)	15 (6)	0.1	10 (9)	8 (3)	0.2

Missing data: 4 cases missing location, 2 cases were additionally missing ethnicity. There was a small amount of missing data: 4 missing rurality; 2 missing ethnicity indicator; 15 missing systolic blood pressure; 2 missing pulse.

*GCS*, Glasgow Coma Score; *ISS*, Injury Severity Score; *bpm*, beats per minute; *mm Hg*, millimetres of mercury.

### Main Results

Table 3 examines differences in EMS infrastructure and transport pathways by incident location alone. Overall, most of the transported cohort (94%) were triaged into the fastest dispatch response (“purple-red”), were transported directly to their highest level of care achieved during the care episode (82%), and were attended, on scene, by the highest practice level of ICP (74%) (Table 3). Single-vehicle crew attendance was uncommon, occurring in 12% of attended patients. Overall, a lower proportion of patients injured rurally were directly transported to the highest level of care achieved in the care episode (77% vs 87% of urban patients) (Table 3). Patients in rural areas took longer to reach in-hospital care (44% vs 7%, out-of-hospital time ≥ 113 minutes, *P* < 0.001). Rural patients had significantly lower theoretical access to healthcare with 60 minutes (2% vs 40%, *P* < 0.001) and a higher level of air transport (51% vs 4% of urban patients, *P* < 0.001).

**Table 3.** Emergency medical services infrastructure and transport pathways, total and by incident location (n = 692).

		Incident location		
	Total (692)^*^	Rural (324)	Urban (364)	
	n (%)	n (%)	n (%)	*P*-value
EMS infrastructure and practice level				
Intensive care paramedic	513 (74.1)	240 (74.1)	269 (73.9)	1
Single crew attendance	87 (12.5)	38 (12.5)	49 (14.5)	0.4
EMS transport pathways				
Fastest dispatch response	654 (94.5)	310 (95.7)	340 (93.5)	0.2
Direct transport to definitive care^*^	572 (82.6)	251 (77.5)	318 (87.4)	0.001
Transport involved air ambulance	183 (26.4)	166 (51.2)	16 (4.4)	<0.001
First attended L1 hospital	385 (55.6)	163 (50.3)	220 (60.4)	0.009
L1 definitive care^*^ hospital	469 (67.8)	217 (66.9)	249 (68.4)	0.6
Theoretical access < 60 minutes	162 (23.4)	16 (1.9)	146 (40.1)	<0.001
Total time to reach hospital (slowest tertile)	173 (25.0)	145 (44.8)	28 (7.7)	<0.001

Missing data: 4 cases missing location.

* Highest level of hospital care achieved during the care episode; ^∧^ slowest tertile of times, lower boundary 113 minutes; all percentages are calculated as column percentages.

*EMS*, emergency medical services; *L1*, Level 1.

Table 4 examines the intersectional differences between incident location and ethnicity. Ethnic differences in EMS transport pathways to hospital-level care were most evident for rural Māori patients. Compared to rural non-Māori a lower proportion of rural Māori received the fastest triaged dispatch (92% Māori vs 97% non-Māori, *P* = 0.05), first attended a tertiary trauma hospital (33% vs 56%, *P* < 0.001), or reached a tertiary trauma hospital (51% vs. 73%, *P* < 0.001). The total out-of-hospital time to reach the first hospital was, on average, slower for rural Māori with 55% in the slowest tertile of total transport times (ie taking at least 113 minutes, or longer) to reach first hospital, compared with 41% of rural non-Māori patients (*P* = 0.02). There was no evidence of differences in theoretical access <60 minutes (*P* = 0.2) or use of air transportation (*P* = 0.7) by ethnicity for rural patients. Additionally, there was no evidence of substantive significant differences in EMS transport pathways between Māori and non-Māori patients injured in urban locations.

**Table 4.** Emergency medical services infrastructure and transport pathways, by combined incident location and ethnicity (n = 692).

	Combined incident location and ethnicity						
	Rural			Urban			
	Māori (85)	Non-Māori (239)		Māori (109)	Non-Māori (253)	
	n (%)	n (%)	*P*-value	n (%)	n (%)	*P*-value
EMS infrastructure and practice level						
Intensive care paramedic	61 (71.8)	179 (74.9)	0.5	87 (79.8)	180 (71.2)	0.09
Single crew attendance	14 (17.9)	24 (10.6)	0.09	10 (9.6)	39 (16.8)	0.08
EMS transport pathways						
Fastest dispatch response	78 (91.8)	232 (97.0)	0.05	102 (93.6)	236 (93.3)	1
Direct transport to definitive care^*^	62 (72.9)	189 (79.1)	0.2	93 (85.3)	224 (88.5)	0.3
Transport involved air ambulance	45 (52.9)	121 (50.6)	0.7	2 (1.8)	14 (5.5)	0.1
First attended hospital L1	28 (32.9)	135 (56.5)	<0.0001	62 (56.9)	157 (62.1)	0.4
L1 definitive care^*^ hospital	43 (50.6)	174 (72.8)	<0.0001	71 (65.1)	176 (69.6)	0.4
Theoretical access <60 minutes	2 (2.4)	14 (5.8)	0.2	48 (44.0)	98 (38.7)	0.3
Total time to reach hospital (slowest tertile)	47 (55.3)	98 (41.0)	0.02	7 (6.4)	21 (8.3)	0.5

Missing data: 4 cases missing location, 2 cases missing ethnicity.

* Highest level of hospital care achieved during the care episode; ^ slowest tertile of times, lower boundary 113 minutes; all percentages are calculated as column percentages.

*EMS*, emergency medical services; *L1*, Level 1.

As presented in Table 5, some differences in receipt of life-saving EMS interventions were observed by incident location: a greater proportion of rural patients received an EMS intervention (54% rural vs 44% urban, *P* = 0.01). Additionally of those presenting with a life-threatening event, a greater proportion of urban patients received no recorded EMS intervention (5% rural vs. 9% urban, *P* = 0.03). While small percentages they likely reflect the closer proximity of hospital-level care in urban settings. There was no strong evidence of differences in percentages that identified with life-threatening events or that received EMS interventions between Māori and non-Māori in either rural or urban locations.

**Table 5.** Life-threatening problems and potentially life-saving EMS interventions, by incident location and ethnicity (n = 692).

		Incident location		
	Total	Rural	Urban	
	n (%)	n (%)	n (%)	
	n = 692	n = 324	n = 364	*P*-value
Any life-threatening events experienced	115 (16.6)	45 (13.8)	69 (18.9)	0.07
Any potentially life-saving EMS intervention received	338 (48.8)	176 (54.3)	160 (43.9)	0.01
Presence of life-threating event, no EMS intervention	47 (6.8)	15 (4.6)	32 (8.8)	0.03
Life-threatening event	n = 115	n = 45	n = 69	
Airway (A) problem	0 (0.0)	0 (0.0)	0 (0.0)	–
Breathing (B) problem	90 (78.3)	30 (66.7)	59 (85.5)	0.4
Of those with (B), received treatment	46 (51.1)	18 (60.0)	27 (45.8)	0.2
Circulation (C) problem	16 (13.9)	11 (24.4)	4 (5.8)	0.004
Of those with (C), received treatment	8 (50.0)	5 (45.5)	2 (50.0)	0.8
Neurotrauma (N) problem	11 (9.6)	5 (11.1)	6 (8.7)	0.7
Of those with (N), received treatment	1 (<0.0)	1 (<0.0)	0 (0.0)	0.3

Missing data: 4 cases missing location, 2 cases missing ethnicity.

*EMS*, emergency medical services.

For those experiencing life-threatening events the majority experienced breathing problems (78%), with just over half these patients receiving one of the potentially life-saving EMS interventions in (outlined in Figure 1) to address these concerns while out of hospital. Similarly, only half of those with life-threatening circulatory problems (8 of 16) received an identifiable EMS intervention (outlined in Figure 1). There were few substantive differences when examined by incident location (Table 5); however, rural patients were more likely to have a recorded circulatory problem than urban patients (*P* = 0.004). Similarly, there were few intersectional differences by location and ethnicity (results not shown in Table 5) with the exception of life-threatening events, which were more prevalent in urban non-Māori compared to rural non-Māori (13% rural vs 20% urban non-Māori, chi2 = 4.45, *P* = 0.03).

## DISCUSSION

Disparities in EMS transport times in rural located patients are common, and longer EMS transport times are thought to play an important role in survival following major traumatic injury events.^3^^–^^6^ The examination of disparities has largely been limited to rural differences in transport times, however, and there is little known about differential transportation pathways or EMS care received, despite well-known rural and ethnic disparities in major trauma outcomes.^2^ Our study identified considerable differences in EMS response and transport pathways, with these differences patterned by the inter-relationship between the geographical location of the incident and ethnicity. Similar to previous studies, we identified a lower proportion of those injured in rural locations who were directly transported to the highest level of care achieved during the care episode. Similarly, those injured in rural (compared to urban) locations were more likely to take longer to reach first hospital and were more likely to involve air ambulance transportation.^3^^,^^4^^,^^6^

In examining the intersection of geographic location of injury and ethnicity we found overlapping disparities that would not have been identified by examining these sources of disparities individually. Comparisons of rurally located indigenous Māori patients to rural non-Māori patients revealed that despite similar on-scene ISS presentation, rural Māori were triaged to slower dispatch and on-scene response pathways and took longer to reach first hospital. Rural Māori were less likely to reach high-level specialist trauma care and facilities, both as a first hospital or at any time during the episode of care. The opposite was observed for Māori patients injured in an urban location, which were more likely to be prioritised; this may have been due to higher incidence of concussive symptoms identified on scene using the GCS. In combination, these findings suggest that there are additional challenges associated with providing equitable out-of-hospital care for Māori injured in rural locations, potentially set in place by out-of-hospital triaging processes.

To the best of our knowledge this is the first study to describe the inter-relationship between rural and ethnic disparities for out-of-hospital EMS care and transport pathways to hospital-level care following RTC trauma in a national context. Rurally located patients, particularly rurally located Māori patients, were identified as being particularly underserved by out-of-hospital EMS following an RTC, despite similar on-scene presentation. Delays along pathways of care and differences in quality of care resulting in excess Māori mortality have also been identified for rural Māori in other areas of healthcare in NZ, including cancer care.^32^^–^^34^ More specifically, ethnically patterned delays in care have been found for out-of-hospital cardiac arrest (OHCA) in NZ. Māori patients had few EMS-witnessed OHCA and a higher level of bystander intervention, suggesting EMS assistance arrives later or help is not sought immediately, resulting in poorer 30-day survival for Māori patients.^35^ Recent examination of ED processes in NZ also identified delays in care experienced, although a higher proportion of Māori ED presentations are self-presentations (unattended by out-of-hospital EMS) and were triaged to be seen within a longer time frame.^36^

This situation is not unique to NZ. Our study expands upon existing literature regarding health inequities in other countries, especially rural indigenous disparities in Australia, Canada, and the United States. While not specific to EMS many studies of healthcare access and utilisation have found rural location to be a barrier to healthcare that disproportionately affects remote, rural indigenous populations.^37^ Factors presenting as barriers to healthcare for indigenous communities include rural location, communication, cultural differences, and poor access to the positive social determinants of health.^37^^,^^38^ With regard to the provision of emergency trauma care, rural locations present challenges such as long distances and travel times, limited trauma care resources and skilled staff.^39^ Higher mortality rates following traumatic injuries in rural areas have been attributed to longer incident-discovery times, longer out-of-hospital time, limited access to major trauma in-hospital care, and delays in receiving definitive in-hospital care.^3^^,^^5^ Mixed evidence for an intersectional relationship between ‘race’/ethnicity and insurance status has been reported at the level of trauma hospital care in the US healthcare system but has not been examined in the out-of-hospital setting.^2^

Understanding the complex intersectional relationship between the geographic location of injury and ethnicity is important to optimising the planning and targeting of healthcare delivery. The barriers generated by geographical location, such as longer distances and times to travel to centralised tertiary hospital-level care, invariably located in metropolitan centres, are exacerbated by ethnicity. For example, in NZ, Māori are more likely to live in rural and more remote places.^32^ The interweaving of complex systemic and structural factors, including institutional and interpersonal racism, differential distribution of the social determinants of health, less access to specialist care, and longer and slower pathways through health systems, all underpinned by the process of colonisation, are well recognised to generate health inequities.^11^^,^^40^ National healthcare reforms currently underway in NZ are strongly focused on addressing inequities for quality improvements in the healthcare system.^41^ Our findings suggest that addressing the overlap between rural and ethnic disparities through strong, equity-focussed planning and prioritisation and through increased investment in rural services has the potential to improve the delivery of rural EMS for both indigenous and non-indigenous populations.

Achieving equitable healthcare is a persistent challenge for healthcare systems worldwide. Our findings suggest the need for better resourcing of rural EMS service with particular attention to inequities experienced by rural Māori communities. Greater recruitment and training of Māori EMS professionals would address Māori under-representation amongst professional EMS staff and reduce hesitancy in accessing unrepresentative services, as well as reduce patient experiences of institutional and interpersonal racism in NZ healthcare.^42^^–^^44^ Qualitative analyses with EMS professionals are also required to understand ethnic and rural differences in coverage of EMS services, infrastructure, staffing, training, experience, skill levels, and deployment for rural communities. It is important that this includes the perspectives of Māori and rural EMS staff and patient voices. To understand ethnic barriers to accessing care following trauma further research should also include a Māori-led investigation of the continuum of trauma care from out-of-hospital EMS dispatch triage through to access to post-hospital rehabilitation services, including any differences between rural and urban care.

Our study found EMS triaging processes (especially for prioritisation of EMS transport from the scene to a L1 hospital) was comparatively slower for rural Māori patients compared to rural non-Māori. Triaging policy is a further mechanism to address disparities in EMS transport pathways and access to tertiary-level trauma care by potentially providing opportunities to prioritise based on location of incident and ethnicity, alongside life-threatening presentations. Further examination of the reasons for differences in triaging and selection of destination hospital are needed given that cultural differences in communication and interpretations of presenting symptoms have been found to influence access to healthcare in indigenous populations.

Patient/family proximity requests are common reasons for hospital selection in other contexts.^4^^,^^37^ Whānau (family) support for patients in hospital is critical for Māori to mitigate against consistently reported negative hospital experiences.^45^ Recent examination of hospitalisations for Māori identified the difficulties for the provision of whānau support during a hospital transfer, or an away-from-home hospital admission, and it is possible this may influence decisions on destination hospital in situations where a choice exists.^46^ Adherence to New Zealand’s 2017 Out-of-Hospital Major Trauma Triage Policy is being examined in more detail to identify unwarranted clinical variations in transporting EMS patients in this cohort.^47^

The question remains whether the difference in EMS transport and access to tertiary-level trauma care and facilities leads to poorer mortality outcomes following an RTC, requiring further examination. Analysis of the wider cohort including non-transported patients identified that when compared with the non-indigenous NZ population Māori were disproportionately represented amongst on-scene fatalities due to RTC. This finding suggests that along with improved EMS healthcare response following trauma there must be a corresponding effort strengthening primary prevention policies and actions focused on addressing upstream risk factors for RTC, including the social and economic determinants of health.

This study has many strengths beyond examining the intersection between geography and ethnicity relevant to healthcare delivery. The use of a consistent mechanism of injury (in this case vehicle occupants in RTCs), allowed for the examination of rural and ethnic differences within a cohort with a more consistent case mix and injury circumstance between sub-groups. Additionally, this study utilised the rurality of the location of injury incident, which is more closely aligned to EMS need than patient residence. The provision of many health services is planned on the distribution of the usually resident population, which misses the highly mobile nature of a population and the occurrence of injury in locations away from domicile, especially RTC.^48^ Road EMS resourcing in NZ is based on the use of retrospective data to model predicted demand according to dispatch response category, number of incidents in a geographic area, and specified response times using specialist modelling software.

Future EMS placement should also include rurality, ethnicity, and deprivation in order to optimise service coverage. Rural community health needs, including access to health services, are often overlooked, especially for rural Māori and for isolated communities, and this study can inform Priority 3 (focused on placing health services closer to rural communities) of the NZ Rural Health Strategy acknowledging the need to consider placement of EMS services in relation to where rural communities live as well as locations with high occurrence of RTC.^49^ The utilisation of an urban/rural geographic classification specifically developed for use in health policy and research, reduces the likelihood of geographic misclassification.^23^ Finally, the universal free-of-cost access to EMS for trauma care in NZ minimises any selection biases caused by economic factors.

## LIMITATIONS

There are several limitations to this study. We analysed data corresponding to EMS care delivered in NZ between 2016–2018, which therefore may not reflect current EMS practice or destination policies^50^ or be directly generalisable to other countries. The findings are limited to Road and Air EMS captured in ePRF data potentially underestimating EMS use when Air EMS service utilisation is not captured by ePRF data. Reasons for Air EMS activation or non-activation are not available in ePRF data. Previously self-presentation to EDs (ie, walk-ins) has been reported to be more common in Māori patients (63% compared with 57% of non-Māori presentations) thus this study that analysed patients attended by EMS may not be representative of ethnic difference in the incidence of major trauma.^37^ Misclassification of ethnicity occurs for Māori, estimated at a 16% undercount using ethnicity reported by the National Health Index, potentially underestimating differences for Māori.^51^

Analyses are limited to those injured as vehicle occupants in RTCs, and patterns of EMS care and pathways to transport may differ for other injury contexts. Analyses examining differences in EMS interventions delivered involved small numbers of patients limiting the ability to make inferences about observed differences. Results highlight comparisons with *P* < 0.01 or smaller, allaying concerns about false positives with multiple comparisons. The adapted measure of life-threatening events identifies airway, breathing, circulatory, or neurotrauma problems and will, therefore, not capture all critical events; one such example is a ruptured spleen or severe head injury, such as haemorrhage, not immediately indicated by on-scene measurements.

## CONCLUSION

This study identified several disparities in EMS transport pathways that are strongly intertwined with rurality and ethnicity. These findings provide an evidence base to help guide clinical and policy decision-makers in identifying opportunities to optimise the delivery of EMS care and to reduce overlapping disparities associated with EMS care, nationally and internationally. Greater equity-focused planning and investment in rural EMS services to reduce documented disparities in EMS triage, transport. and access to high quality specialist trauma care is clearly warranted and would benefit both indigenous and non-indigenous populations.

## DECLARATIONS

### Ethics Approval and Consent to Participate

The Health and Disability Ethics Committee (reference 18NTB142) provides ethics approval. Access approvals to study datasets was obtained from the New Zealand Trauma Registry (NZTR) Data Governance Group; Ministry of Health for extracts from the National Health Index database; and from St John and Wellington Free Ambulance for EMS data.

### Availability of Data and Materials

The raw data that supports the findings of this study are available from Hone Hato St John, Wellington Free Ambulance and the NZTR. Restrictions apply to the availability of this data. Derived data is available from the corresponding author on request.
